# Supplementary material for: A fungal pathogen deploys a small silencing RNA that attenuates mosquito immunity and facilitates infection
Source: Nat Commun. 2019 Sep 20;10:4298. doi: 10.1038/s41467-019-12323-1 (PMC6754459; doi:10.1038/s41467-019-12323-1)
Supplement: Supplementary file 1 — Supplementary information [file 41467_2019_12323_MOESM1_ESM.pdf]

# Supplementary Information

## A fungal pathogen deploys a small silencing RNA that attenuates mosquito immunity and facilitates infection

Chunlai Cui, Yan Wang, Jingnan Liu, Jing Zhao, Peilu Sun, Sibao Wang

Corresponding authors: Wang, S. (sbwang@sibs.ac.cn)

**a**

| Name | Sequence (5'→3') | Length (nt) | 36h (Reads) | 60h (Reads) | 84h (Reads) |
|---|---|---|---|---|---|
| bba-milR1 | UGACUGGCUGUUAAUUGACUGG | 22 | 8 | - | - |
| bba-milR2 | AACACCGGGUGCUGUACAU | 19 | 47 | - | - |
| bba-milR3 | UCGAGAGUAGGACAAUUGUUA | 21 | - | - | 5 |
| bba-milR4 | AGAAGAUUAGCAUGGCCCCUG | 21 | - | - | 18 |

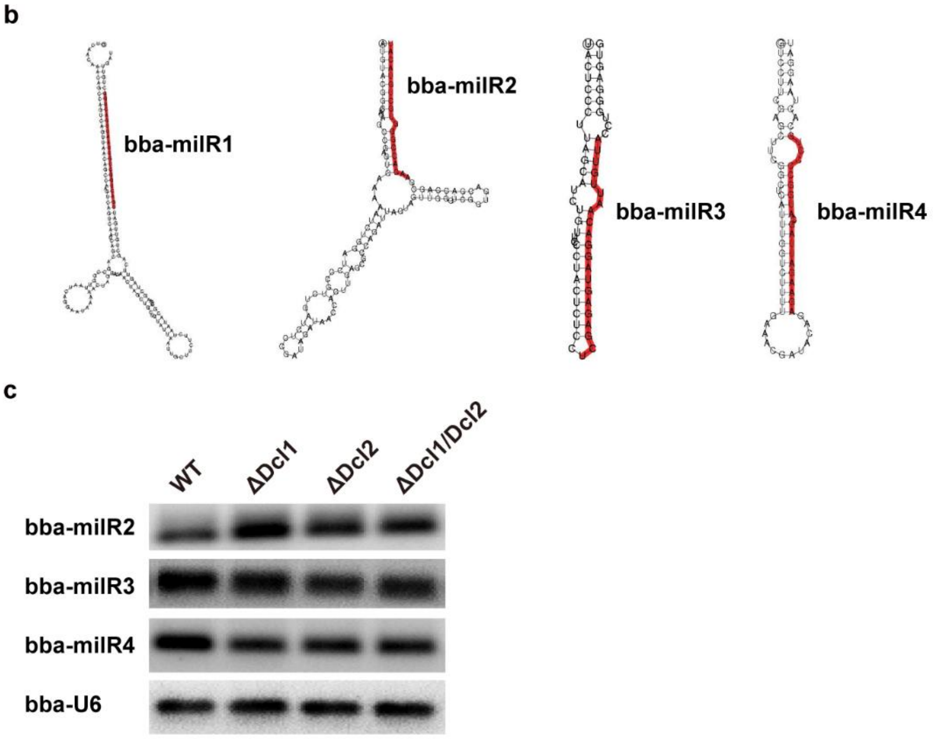


**Supplementary Figure 1**

**Sequences and predicted secondary structures of *Beauveria bassiana* miRNA-like RNAs (milRNAs)**.

(a) Sequences, length and reads of four milRNAs identified in sRNA libraries from *B. bassiana*-infected mosquitoes. (b) Predicted secondary structure of the bba-milR1, bba-milR2, bba-milR3 and bba-milR4 precursors (pre-milRNAs). The mature milRNAs are highlighted in red. (c) bba-milR2, bba-milR3 and bba-milR4 can be detected in WT, *Dicer1* deletion mutant (ΔDcl1), *Dicer2* deletion mutant (ΔDcl2) and *Dicer1/Dicer2* double mutant (ΔDcl1/Dcl2). Source data are provided as a Source Data file.

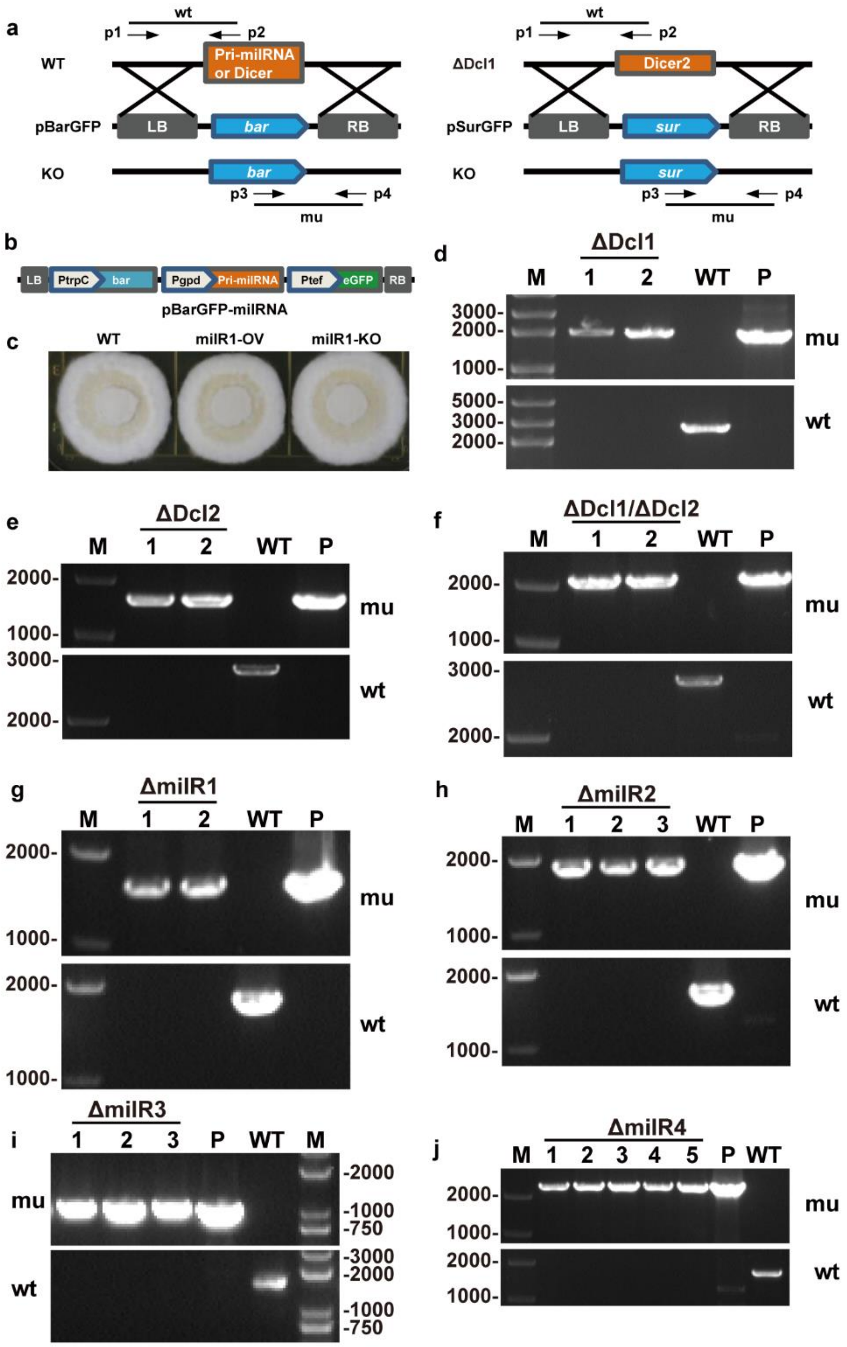


**Supplementary Figure 2**

**Knockout or over-expression of milRNA genes and *Dicer* in *B. bassiana* RESEF252.**

(a) Schematic diagram of the milRNA gene or *Dicer* wild type locus and the plasmids pBar-GFP and pSur-GFP containing flanking regions of the pre-milRNA or *Dicer*, which were used for milRNA genes or *Dicer* disruption through double crossover recombination. LB, left border; RB,

right border; bar, the Ignite/basta-resistance (bar) gene; sur, the sulfonylurea resistance (sur) gene. Primer pairs p1 and p2 were used to amplify WT genome DNA fragment, p3 and p4 were used to verify knock-out mutants. (b) Schematic diagram of the vector pBarGFP-miRNA that was used for milRNAs overexpression (OV). Pgpd, *Aspergillus nidulans gpdA* promoter; PtrpC, *A. nidulans trpC* terminator; Ptef, *Trichoderma reesei* translation elongation factor 1 alpha gene promoter. (c) Growth and conidia production of milR1-OV and milR1-KO are similar to wild-type strain (WT). Verification of ΔDcl1 (d), ΔDcl2 (e), ΔDcl1/ΔDcl2 (f), ΔmilR1 (g), ΔmilR2 (h), ΔmilR3 (i) and ΔmilR4 (j) mutant strains by PCR using two relevant primer pairs of p1 and p2, p3 and p4. ΔDcl1/ΔDcl2 double mutants were generated by disrupting *Dcl2* in a *Dcl1* mutant strain, and verified by PCR detecting *Dcl2* gene in the putative transformants using the two relevant primer pairs of p1 and p2, p3 and p4 (see right diagram of the panel a). The plasmid pBar-GFP or pSur-GFP was used as a positive control. Source data are provided as a Source Data file.

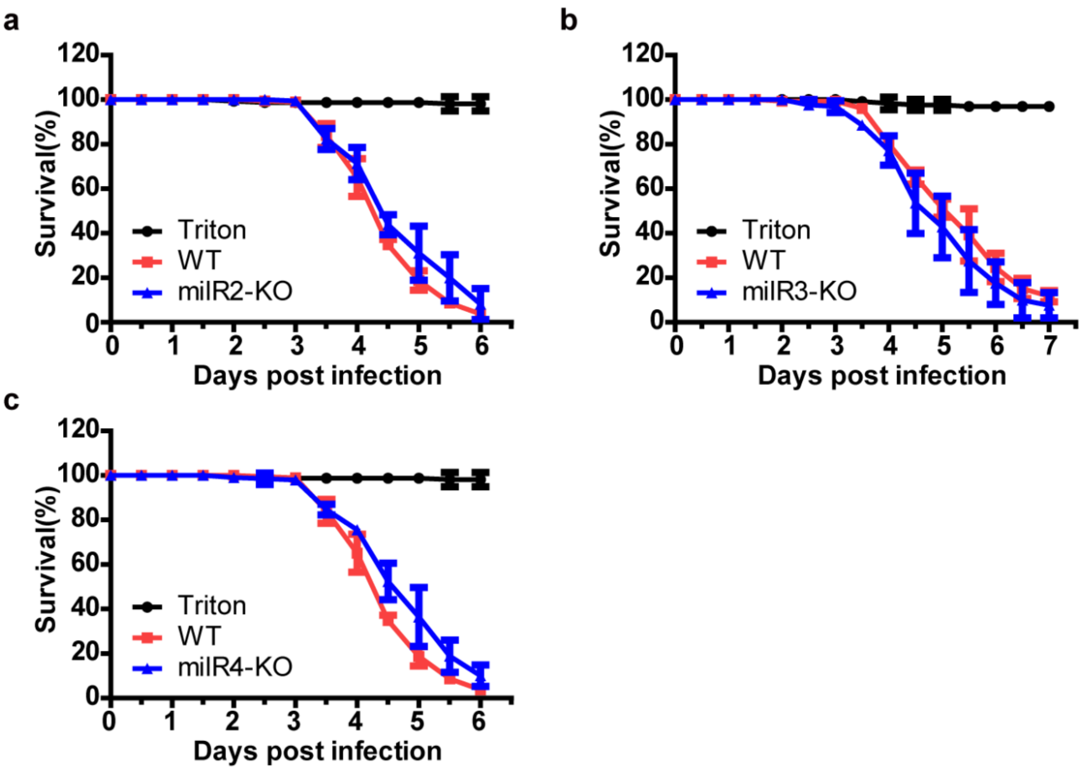


**Supplementary Figure 3**

**Survival of adult female *A. stephensi* mosquitoes after topical infection with suspension of $10^8$ conidia/ml of wild type strain ARSEF252 (WT), Knockout strains bba-milR2-KO (a), milR3-KO (b) and milR4-KO (c).** Survival percentages are means of three biological replicates of 50 mosquitoes each (mean ± s.e.m). The statistical significance of survival curves was analyzed with the log-rank (Mantel-Cox) test. There is no significant difference in the virulence between WT and milR2-KO, milR3-KO or milR4-KO to the mosquitoes. The experiment was repeated three times with similar results. Source data are provided as a Source Data file.

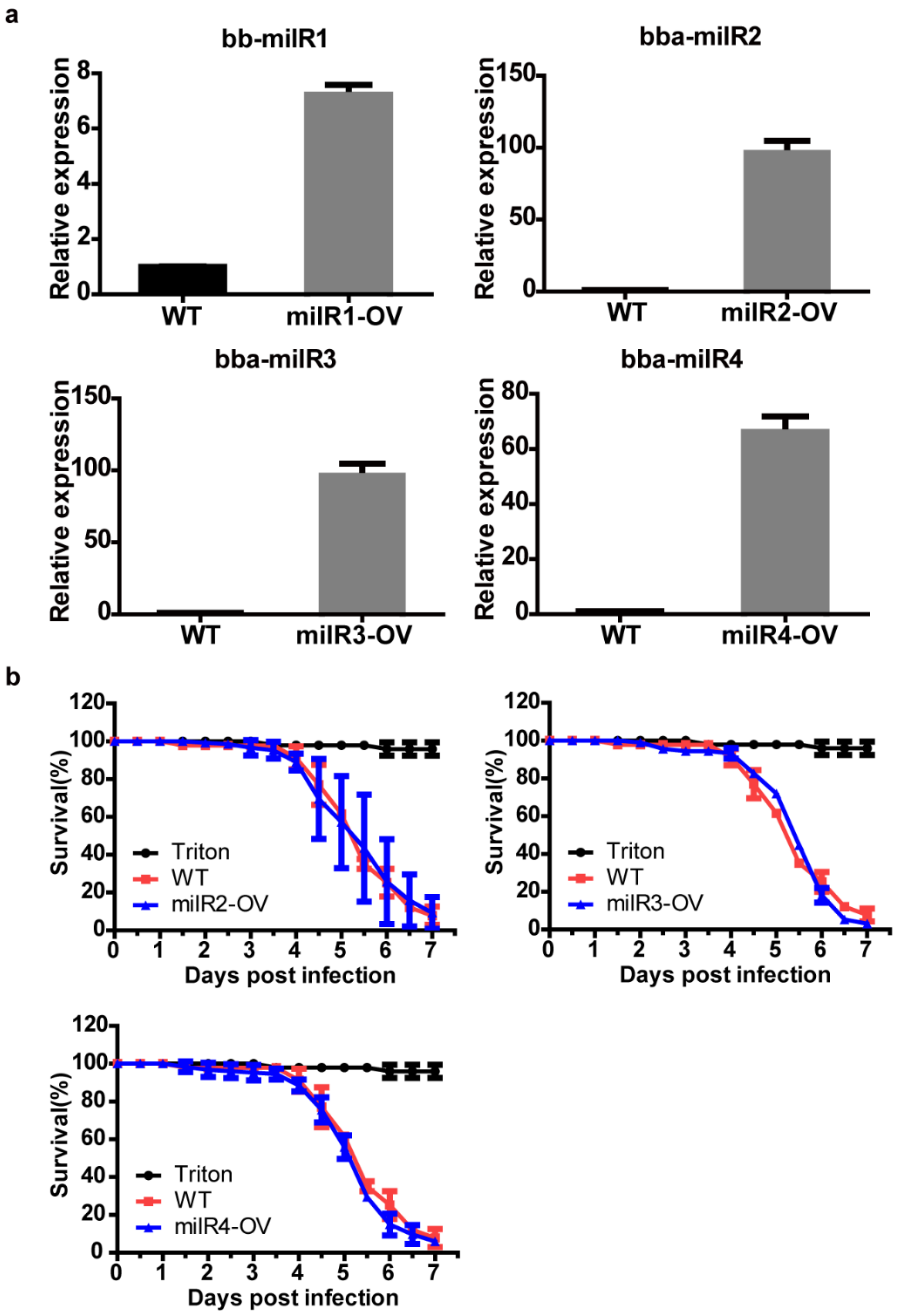


**Supplementary Figure 4**

**Effect of overexpression of bba-milR2, bba-milR3 and bba-milR4 on fungal virulence to adult female *A. stephensi* mosquitoes**.

(a) Analysis of expression of bba-milR1, bba-milR2, bba-milR3 and bba-milR4 relative to U6 in *B. bassiana* ARSEF252 and overexpression strains, as determined by qPCR. The expression values are normalized to WT. (b) Survival curves of female mosquitoes following topical inoculation with suspension of $10^7$ conidia/ml of the wild-type and milR2-OV, milR3-OV and milR4-OV strains. Survival percentages are means of three biological replicates of 50 mosquitoes each (mean ± s.e.m). The statistical significance of survival curves was analyzed with the log-rank (Mantel-Cox) test. There is no significant difference in the virulence between WT and milR2-OV, milR3-OV or milR4-OV against the mosquitoes. Similar results were obtained in three biological repeats. Source data are provided as a Source Data file.

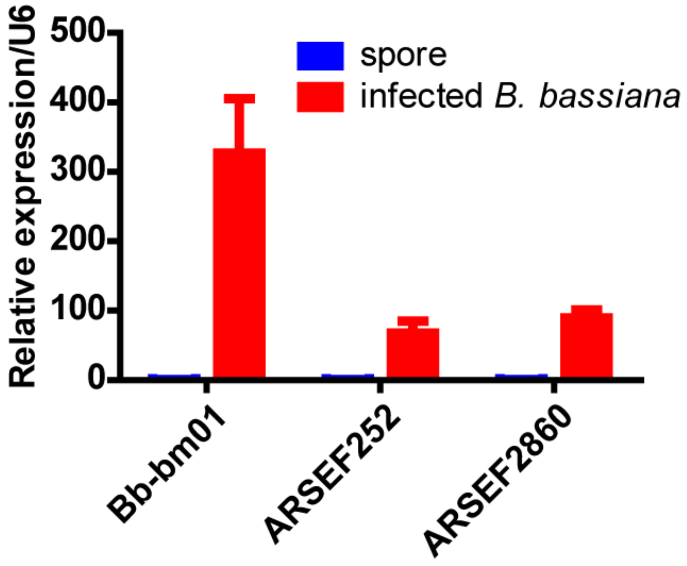


**Supplementary Figure 5**

**bba-milR1 is expressed at 36 h after infection of *A. stephensi* mosquitoes with the *B. bassiana* strains Bb-bm01, ARSEF252 and ARSEF2860**. The *B. bassiana* U6 small nuclear RNA (U6) was used as an internal reference in qRT-PCR assays. The expression values are normalized to spore. The experiment was repeated three times with similar results. Source data are provided as a Source Data file.

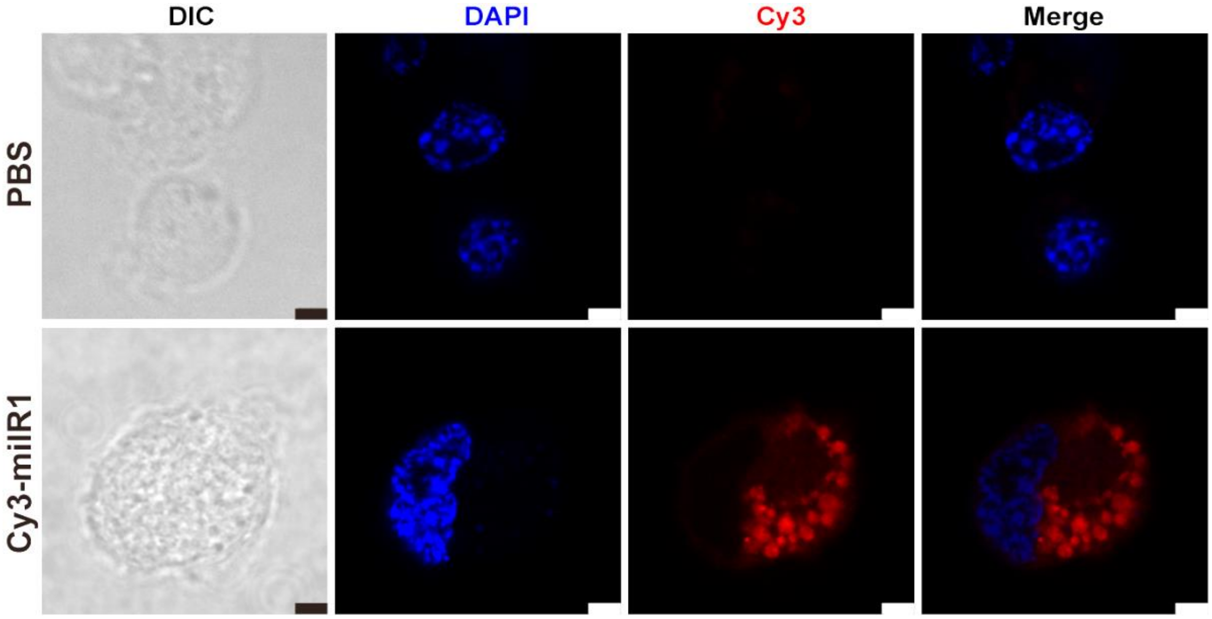


**Supplementary Figure 6**

**bba-milR1 sRNAs are translocated into cultured *Drosophila* cells via vesicles.**

bba-milR1 was translocated into *Drosophila* cells via vesicles. Cy3-labelled bba-milR1 (2 μM) was added in *Drosophila* S2 cell cultures for 24 h, then washed with PBS, fixed in 4% paraformaldehyde and stained with DAPI. DIC, differential interference contrast microscopy; Red, Cy3; blue, DAPI. Scale bars, 3 μm.

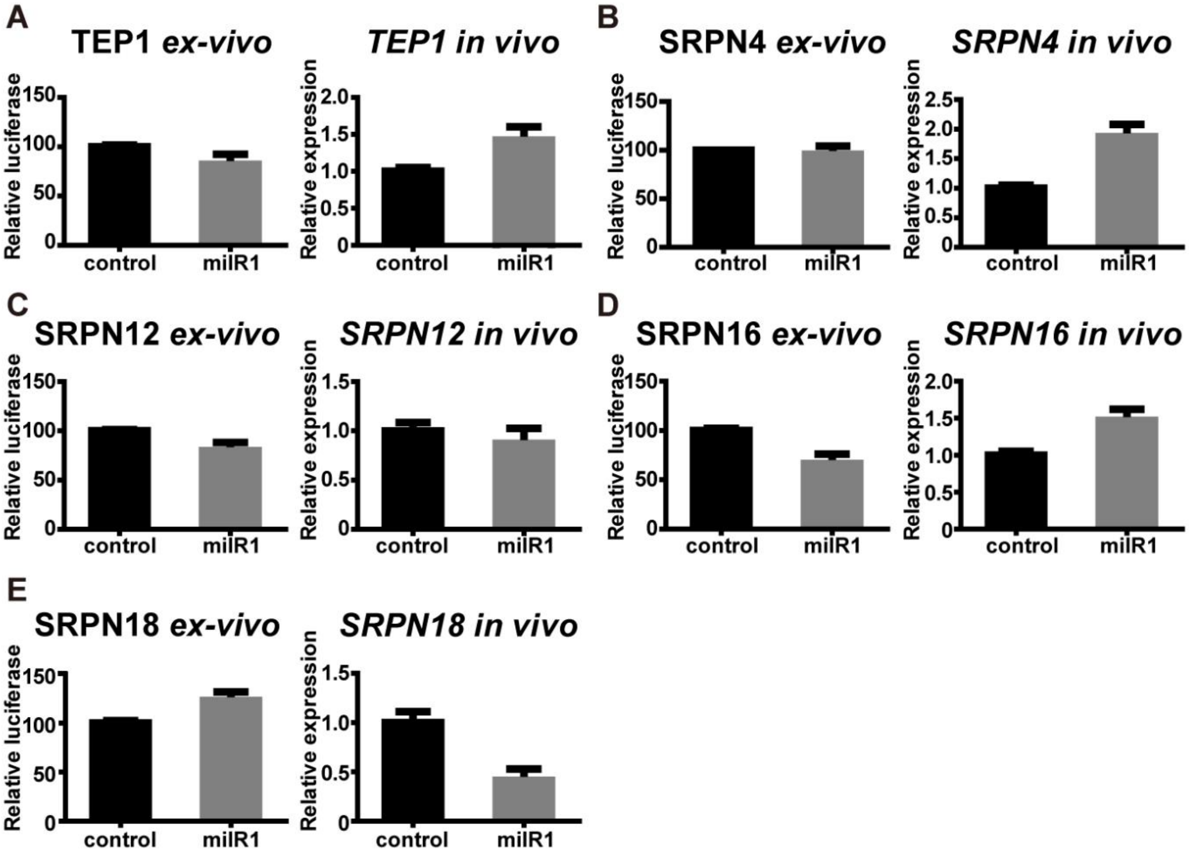


**Supplementary Figure 7**

**Verification the predicted targets of bba-milR1 *ex-vivo* and *in vivo*.**

The candidate target genes were separately verified *ex-vivo* in HEK293T cells and *in vivo* in *Anopheles* mosquitoes. (a) bba-milR1 down-regulated the expression of TEP1 *ex-vivo*, but up-regulated the expression of *TEP1 in vivo*. (b) bba-milR1 down-regulated the expression of SRPN4 *ex-vivo*, but up-regulated the expression of *SRPN4 in vivo*. (c) bba-milR1 down-regulated the expression of SRPN12 *ex-vivo*, but had no effect on the expression of *SRPN12 in vivo*. (d) bba-milR1 down-regulated the expression of SRPN16 *ex-vivo*, but up-regulated the expression of *SRPN16 in vivo*. (e) Expression of SRPN18 was up-regulated *ex-vivo* but down-regulated *in vivo* by bba-milR1. The experiments were repeated three times with similar results. The expression values are normalized to control. Source data are provided as a Source Data file.

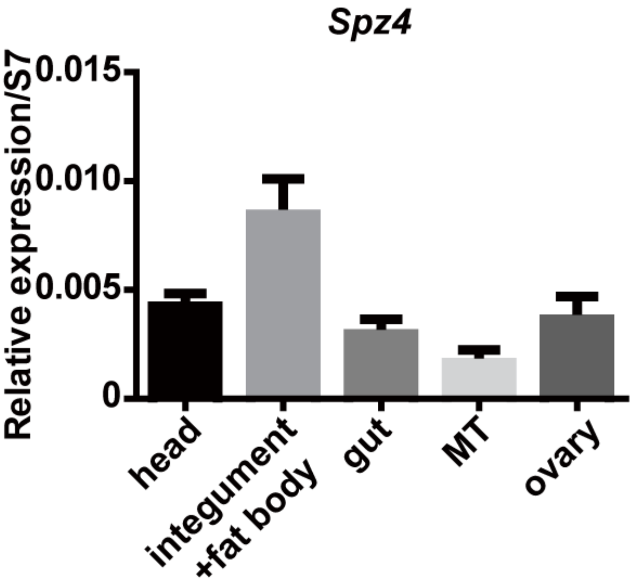


**Supplementary Figure 8**

**Expression patterns of *Spz4* in different tissues of the adult female *A. stephensi* mosquito**.

MT, Malpighian tubule. Similar results were obtained in three biological repeats. Source data are provided as a Source Data file.

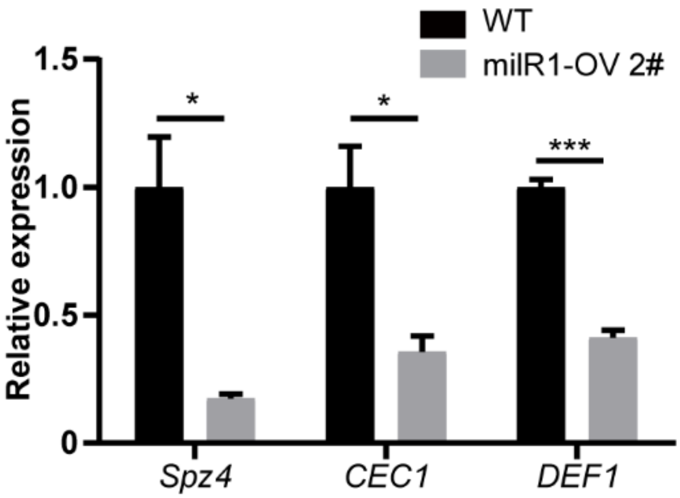


**Supplementary Figure 9**

**bba-milR1 suppresses mosquito innate immunity by down-regulating expression of *Spz4*.**

The relative transcript levels of *Spz4*, *cecropin 1* (*CEC1*) and *defensin 1* (*DEF1*) in mosquitoes at 48 h post infection (hpi) with WT and milR1-OV 2#. The expression values are normalized to WT. The results confirm the phenotypes shown in Fig. 4. * $P < 0.05$, *** $P < 0.001$. $P$ value < 0.05 was regarded as statistically significant (Student's $t$ test). Source data are provided as a Source Data file.

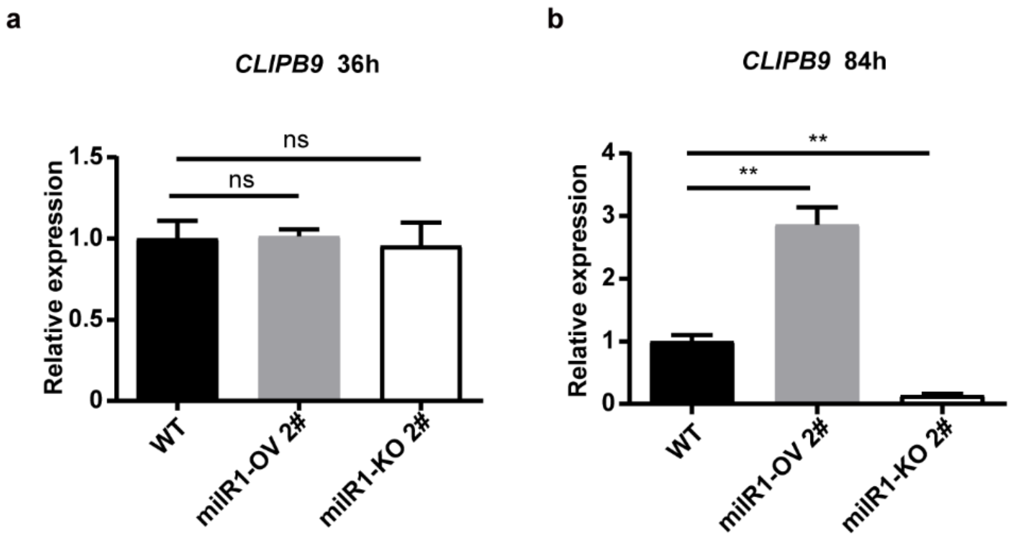


**Supplementary Figure 10**

**Bba-milR1 up-regulates *CLIPB9* expression at the later infection stage.**

*CLIPB9* transcription levels during infection progress with WT, milR1-OV 2# and milR1-KO 2# strains. (a) The relative transcript levels of *CLIPB9* in mosquitoes at 36 h post infection with WT, milR1-OV or milR1-KO strains. There was no significant difference in *CLIPB9* expression among the groups at this time point. (b) The relative transcript levels of *CLIPB9* in mosquitoes at 84 h post infection with WT, milR1-OV 2# or milR1-KO 2# strains. The expression values are normalized to WT. The results confirm the phenotypes with the strains milR1-OV 1# and milR1-KO 1# shown in Fig. 5. ** $P < 0.01$ was regarded as statistically significant (Student's *t* test). Source data are provided as a Source Data file.

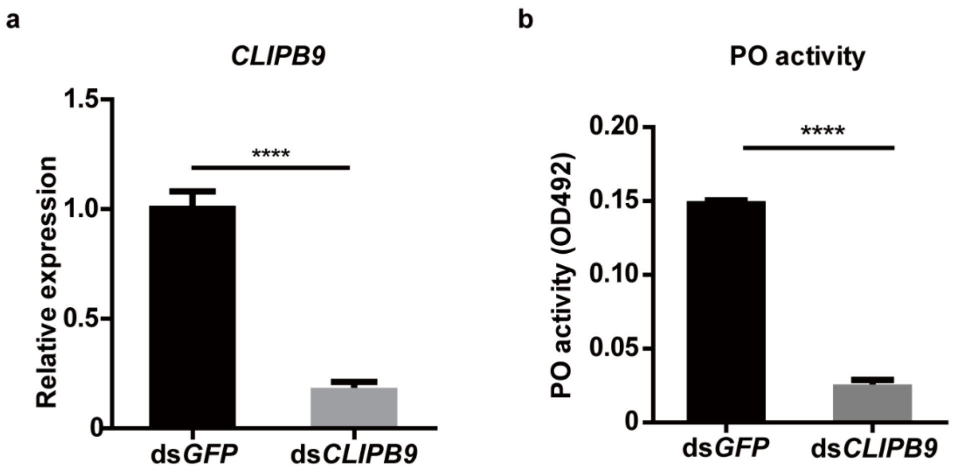


**Supplementary Figure 11**

**CLIPB9 modulates the PO activity of *A. stephensi* hemolymph.**

(a) Analysis of silencing efficiency of *CLIPB9* in adult female *A. stephensi* mosquitoes. Systemic injection of *CLIPB9* dsRNA (dsCLIPB9) significantly reduced *CLIPB9* transcript levels. The expression values are normalized to ds*GFP*. (b) Silencing of *CLIPB9* results in significant decrease in *A. stephensi* hemolymph PO activity. Values are mean ± s.e.m. The experiments were repeated three times with similar results. ****$P < 0.0001$ (Student's *t* test). Source data are provided as a Source Data file.

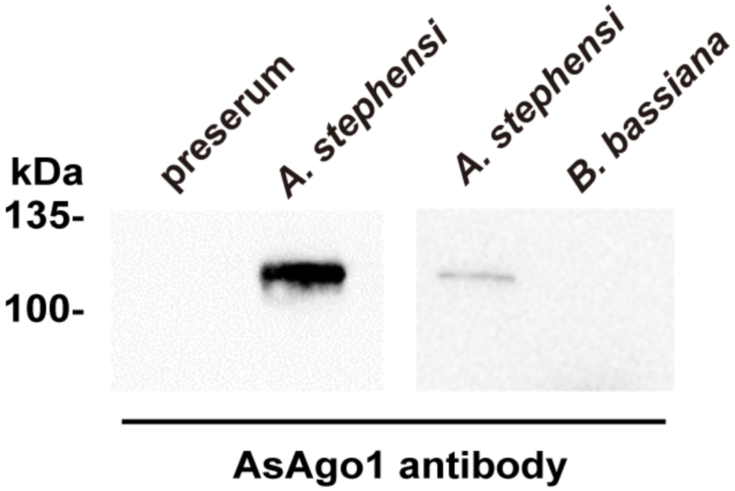


**Supplementary Figure 12**

**Specificity analysis of AsAGO1 antibody**.

The protein sample of *Anopheles stephensi* and *Beauveria bassiana* were applied to western-blot analysis. AsAGO1 antibody recognizes ~110kD band in the protein sample of *A. stephensi*, but not with *B. bassiana* proteins. The preimmune serum of rabbit was used as the negative control. Source data are provided as a Source Data file.

**Supplementary Table 1 │ bba-milR1 target prediction in *Anopheles stephensi* mosquito using the multi-algorithm and multi-model strategy.**

<table>
<tr><th>Gene ID</th><th>Gene Name</th><th>mfe</th><th>Target Site</th></tr>
<tr><td>ASTE010227</td><td>TEP1</td><td>-16.8 kcal/mol</td><td>miRNA: 3' GGTCAGTTAATTGTCGGTCAGT 5'<br>| | | || |||||||<br>Target: 5' CGAACCTACTCAC-GCCAGTCA 3'</td></tr>
<tr><td>ASTE011729</td><td>Spz4</td><td>-22.9 kcal/mol</td><td>miRNA: 3' GGTCAGTTAATTGTCGGTCAGT 5'<br>|| ||| |||||||<br>Target: 5' GCCCAGAAAGAACCGCCAGTCG 3'</td></tr>
<tr><td>ASTE002770</td><td>CLIPB9</td><td>-22.8 kcal/mol</td><td>miRNA: 3' GGTCA-GTTAATTGTCGGTCAGT 5'<br>|| | :| | |:|||||||<br>Target: 5' GTTGTACCGTCACCGGCCAGTCC 3'</td></tr>
<tr><td>ASTE010655</td><td>SRPN4</td><td>-22.3 kcal/mol</td><td>miRNA: 3' GGTCAGTTAA--TTGTCGGTCAGT 5'<br>|| ||| | ||||||||||<br>Target: 5' ACAG-CAACTGCAACAGCCAGTAC 3'</td></tr>
<tr><td>ASTE008874</td><td>SRPN12</td><td>-24.2 kcal/mol</td><td>miRNA: 3' GGTCAGTTAATTGTCGGTCAGT 5'<br>:|| |||:|||||||<br>Target: 5' AACACGGATGAACGGCCAGTCA 3'</td></tr>
<tr><td>ASTE001474</td><td>SRPN16</td><td>-21.3 kcal/mol</td><td>miRNA: 3' GGTCAGTTAATTGTCGGTCAGT 5'<br>|| |: | : |||||||<br>Target: 5' GCAGCCGTCTTCTCGCCAGTCA 3'</td></tr>
<tr><td>ASTE001727</td><td>SRPN18</td><td>-23.4 kcal/mol</td><td>miRNA: 3' GGTCAGTTAATTG-TCGGTCAGT 5'<br>|| |:| | ||||||||<br>Target: 5' CCAGACGACGCTCGAGCCAGTCG 3'</td></tr>
</table>
