# Supplementary material for: A fungal pathogen deploys a small silencing RNA that attenuates mosquito immunity and facilitates infection
Source: Nat Commun. 2019 Sep 20;10:4298. doi: 10.1038/s41467-019-12323-1 (PMC6754459; doi:10.1038/s41467-019-12323-1)
Supplement: Supplementary file 3 — Description of Additional Supplementary Files [file 41467_2019_12323_MOESM3_ESM.pdf]

Description of Additional Supplementary Files

File Name: Supplementary Data 1
Description: Primers used in this study.
