# Supplementary material for: A fungal pathogen deploys a small silencing RNA that attenuates mosquito immunity and facilitates infection
Source: Nat Commun. 2019 Sep 20;10:4298. doi: 10.1038/s41467-019-12323-1 (PMC6754459; doi:10.1038/s41467-019-12323-1)
Supplement: Supplementary file 5 — Reporting Summary [file 41467_2019_12323_MOESM5_ESM.pdf]

nature research

Corresponding author(s): Sibao Wang

Last updated by author(s): Aug 23, 2019

Reporting Summary

Nature Research wishes to improve the reproducibility of the work that we publish. This form provides structure for consistency and transparency in reporting. For further information on Nature Research policies, see Authors & Referees and the Editorial Policy Checklist.

Statistics

For all statistical analyses, confirm that the following items are present in the figure legend, table legend, main text, or Methods section.

n/a	Confirmed	
☐	☒	The exact sample size (*n*) for each experimental group/condition, given as a discrete number and unit of measurement
☐	☒	A statement on whether measurements were taken from distinct samples or whether the same sample was measured repeatedly
☐	☒	The statistical test(s) used AND whether they are one- or two-sided<br>*Only common tests should be described solely by name; describe more complex techniques in the Methods section.*
☒	☐	A description of all covariates tested
☒	☐	A description of any assumptions or corrections, such as tests of normality and adjustment for multiple comparisons
☐	☒	A full description of the statistical parameters including central tendency (e.g. means) or other basic estimates (e.g. regression coefficient) AND variation (e.g. standard deviation) or associated estimates of uncertainty (e.g. confidence intervals)
☐	☒	For null hypothesis testing, the test statistic (e.g. *F*, *t*, *r*) with confidence intervals, effect sizes, degrees of freedom and *P* value noted<br>*Give P values as exact values whenever suitable.*
☒	☐	For Bayesian analysis, information on the choice of priors and Markov chain Monte Carlo settings
☒	☐	For hierarchical and complex designs, identification of the appropriate level for tests and full reporting of outcomes
☒	☐	Estimates of effect sizes (e.g. Cohen's *d*, Pearson's *r*), indicating how they were calculated

Our web collection on statistics for biologists contains articles on many of the points above.

Software and code

Policy information about availability of computer code

Data collection	N/A
Data analysis	miRCat2(http://srna-workbench.cmp.uea.ac.uk/mircat2/), PITA (version 6), DESeq2 (version 1.14.1), miRanda (version aug2010), microtar (version 0.9.6), GraphPad Prism (version 5.00)

For manuscripts utilizing custom algorithms or software that are central to the research but not yet described in published literature, software must be made available to editors/reviewers. We strongly encourage code deposition in a community repository (e.g. GitHub). See the Nature Research guidelines for submitting code & software for further information.

Data

Policy information about availability of data

All manuscripts must include a data availability statement. This statement should provide the following information, where applicable:

- Accession codes, unique identifiers, or web links for publicly available datasets
- A list of figures that have associated raw data
- A description of any restrictions on data availability

All data needed to understand and access the conclusions of this research are available in the main text and supplementary materials. The sequencing datasets were deposited in the National Center for Biotechnology Information Sequence Read Archive (accession: PRJNA517599).

Field-specific reporting

Please select the one below that is the best fit for your research. If you are not sure, read the appropriate sections before making your selection.

☒ Life sciences ☐ Behavioural & social sciences ☐ Ecological, evolutionary & environmental sciences

For a reference copy of the document with all sections, see nature.com/documents/nr-reporting-summary-flat.pdf

Life sciences study design

All studies must disclose on these points even when the disclosure is negative.

Sample size	No statistical method was used to pre-determine sample sizes. Samples sizes for small RNA-seq were about 50 infected mosquitoes from two biological replicates per time point. Sample sizes for qRT-PCR were 3 biological replicates, and were chosen to achieve reasonable estimates of variation.
Data exclusions	No data were excluded
Replication	All replications gave similar data
Randomization	Mosquitoes of same eclosion time were separated into different groups randomly in bioassay test.
Blinding	Blinding was not relevant to this study.

Reporting for specific materials, systems and methods

We require information from authors about some types of materials, experimental systems and methods used in many studies. Here, indicate whether each material, system or method listed is relevant to your study. If you are not sure if a list item applies to your research, read the appropriate section before selecting a response.

Materials & experimental systems

n/a	Involved in the study	
☐	☒	Antibodies
☐	☒	Eukaryotic cell lines
☒	☐	Palaeontology
☒	☐	Animals and other organisms
☒	☐	Human research participants
☒	☐	Clinical data

Methods

n/a	Involved in the study	
☒	☐	ChIP-seq
☒	☐	Flow cytometry
☒	☐	MRI-based neuroimaging

Antibodies

Antibodies used	Ago1 antibody was a custom-made antibody by GenScript company.
Validation	AsAGO1 antibody specificity was validated in Supplementary Figure 12.

Eukaryotic cell lines

Policy information about cell lines

Cell line source(s)	Aedes albopictus C6/36 cells (ATCC), Homo sapiens 293T (ATCC), Drosophila melanogastor S2 cells (Thermo Fisher Scientific).
Authentication	No authentication has been used.
Mycoplasma contamination	Cell lines were not contaminated by mycoplasma.
Commonly misidentified lines (See ICLAC register)	No cell lines used are listed in the database of commonly misidentified cell lines.
